# Property of Modified Bovine Bone Glue as an Environmental Additive in Water-Based Drilling Fluids

**DOI:** 10.1021/acsomega.4c01000

**Published:** 2024-03-25

**Authors:** Weichao Du, Bingqian Song, Xianbin Huang, Gang Chen

**Affiliations:** † Shaanxi Province Key Laboratory of Environmental Pollution Control and Reservoir Protection Technology of Oilfields, Shaanxi University Engineering Research Center of Oil and Gas Field Chemistry, Xi’an Shiyou University, Xi’an 710065, China; ‡ Shandong Key Laboratory of Oilfield Chemistry, China University of Petroleum (East China), Qingdao 266580, China

## Abstract

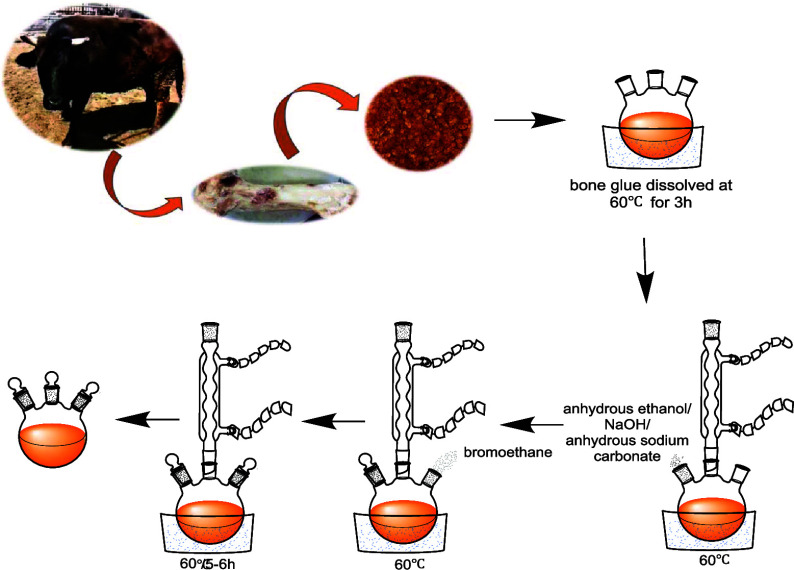

At present, animal bone glue (BG) is being widely used in many fields, but there are no studies reported on oilfield chemistry. In this paper, an environmental water-based drilling fluids additive named bromoethane-modified bone glue (BG) was developed by using bovine bone glue and bromoethane as raw materials, anhydrous ethanol as solvent, sodium hydroxide as alkaline hydrolysis agent, and sodium carbonate as a system pH regulator. The inhibition, filtration performance, and temperature resistance of BG were evaluated. Performance study results show that the linear swelling rate of sodium bentonite (Na-MMT) was decreased from 50.2% (in tap water) to 38.2% (in 4 wt % BG solutions), and filtration loss was reduced from 30 mL (in tap water) to 12 mL (in 5 wt % BG). Hot-rolling experiments show that the BG solution still exhibits good performance even after 16 h × 130 °C. The reasons for BG to achieve excellent performance were analyzed through scanning electron microscopy (SEM), Fourier transform infrared (FT-IR) spectroscopy, X-ray diffraction (XRD), ζ potential, thermogravimetric analysis (TGA), and microstructure. The results of SEM and FT-IR show that BG can fully dissolve in water and adsorb on the surface of clay particles by relying on its own adsorption functional groups such as −OH and −COOH. When 4% BG was added, ζ potential analysis revealed that the clay particle size declined by 0.502 μm, which indicated that BG can inhibit clay hydration swelling dispersion.

## 1 Introduction

As one of the world’s major energy sources, oil is important for modern industrial development. With the increasing demand for energy and the development of oil drilling technology, deep and ultradeep wells have become important directions for drilling development.^1−3^ In the process of developing deep well oil and gas resources, wellbore instability is a common drilling accident problem in deep well drilling operations, which has seriously affected the exploration process of oilfield resources.^4,5^ During the drilling process, shale inhibitors, filtration agents, and other additives are usually added to drilling fluids to prevent drilling safety accidents such as collapse, sticking, leakage, and blowout.^6−8^

In recent years, with the global petroleum industry’s environmental awareness of drilling fluids gradually increased, the development of environmentally friendly drilling fluid additives became imminent, so more and more researchers around the world have performed a series of studies on environmentally friendly drilling fluid additives from plants in nature.^9−11^ Natural materials including lignin, plant starch, and carboxymethylcellulose (CMC), and *Aloe vera* modified by artificial methods were used as environmentally friendly additives in drilling fluids; the results show that these modified natural materials can improve the performance of drilling fluids to a certain extent.^9,12−14^

For example, Hossain et al. introduced grass into drilling fluids and prepared a water-based drilling fluid using bentonite, grass, and water, and studied the rheological and filtration properties of the drilling fluids; the results indicated that grass samples with different particle sizes and concentrations can improve the viscosity and filtration performance of drilling fluids.^15^ Ma et al. synthesized a novel copolymer using acrylamide (AM), 2-acrylamido-2-methyl-1-propanesulfonic acid (AMPS), diallyl dimethylammonium chloride (DMDAAC), and a synthesized betaine monomer (vinylbenzenesulfonate) as raw materials. The results indicated that the synthesized copolymer exhibits good filtration performance under high-temperature environments containing salt.^16^ Wajheeuddin et al. investigated the performance of three natural materials, date seeds, powdered grass, and grass ash, as additives to a drilling mud system and showed that date seeds, powdered grass, and grass ash can be used as rheology modifiers and that they can control the filtration loss.^17^ Jiang et al. composites of gelatin and inorganic salt (KCl) or organic salt (2,3-epoxypropyl-trimethylammonium chloride, EPTAC) were prepared as environmentally friendly shale inhibitors to perfect the inhibitive effect. The results indicated that gelatin composite with EPTAC shows better synergistic inhibition than that with KCl.^18^

In addition to some natural materials currently used, there is also a natural material called animal bone glue (BG). Animal bone glue is a gelatinous substance extracted from animal bones, cartilage, etc. After odor removal and washing, it can yield a relatively pure animal bone glue.^19,20^ By relevant literature research, animal bone glue was extracted from animal tissues such as cartilage and connective tissue through industrial processing. It is a green, biodegradable natural material that has been widely used many years ago and has an promising future in application.^21−23^

At present, animal bone glue is widely used in the industry, medical treatment, and other fields, and the application has been effective.^24,25^ For example, Yan et al. studied the bone glue/polyurethane composite modified asphalt (CMA) prepared using bone glue, polyurethane, and neat asphalt, and realized its application in road engineering.^26^ Norton et al. studied the properties of bone adhesive and found that the chemical composition of bone adhesive can react in humid environments, forming a strong and durable bond with live bone and metal implants. In clinical practice, this adhesive provides the possibility of immediate and prolonged stability.^27^ Rizvi et al. modified asphalt binders with bone glue to improve the rheological and mechanical properties of asphalt materials. The results indicate that bone glue, as a modifier, not only reduces costs but also improves the long-term performance characteristics of the road surface.^28^

**Figure 1:**
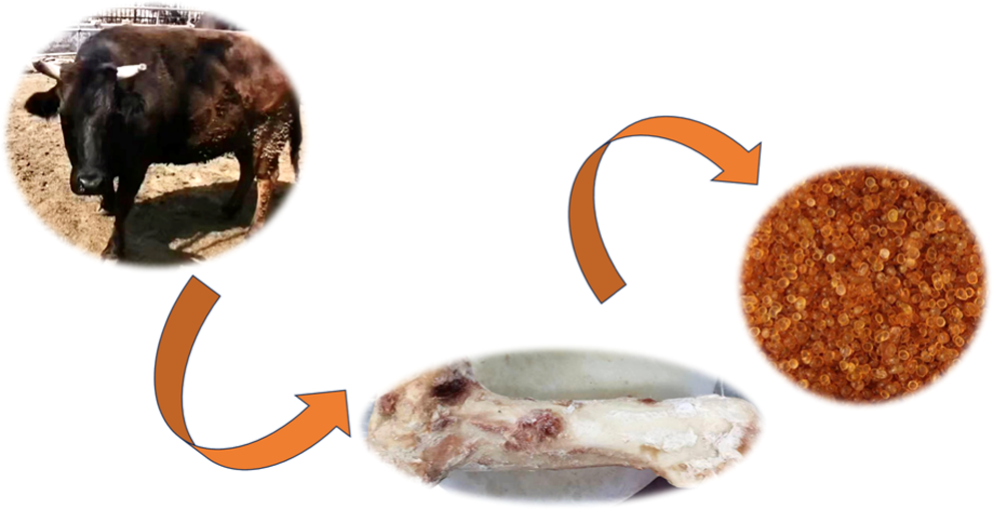
Sources of bovine bone glue.

Given the advantages of low cost, convenient use, and good bonding performance, bone glue has a promising future development prospect and is expected to be applied in drilling fluids. In this paper, BG (Figure 1) was developed using bovine bone glue and bromoethane as raw materials, anhydrous ethanol as solvent, sodium hydroxide as alkaline hydrolysis agent, and sodium carbonate as a system pH regulator, and then the modified bone glue (BG) was applied to drilling fluids and its properties such as filtration and inhibition in drilling fluids were evaluated.

## 2 Materials and Methods

### 2.1 Materials

Bovine bone glue was supplied by Henan Fine Chemicals Factory, which was industrial grade. Bromoethane, sodium hydroxide, and sodium carbonate were obtained from Tianjin Damao Chemical Reagent Factory, and anhydrous ethanol was acquired from Tianjin Tianli Chemical Reagent Co., all of which were analytically pure and their purity was 99%. Calcium-based clay was procured from Xi’an Permanent Chemical Co., Ltd., which belonged to industrial grade.

### 2.2 Synthesis of BG

**Figure 2:**
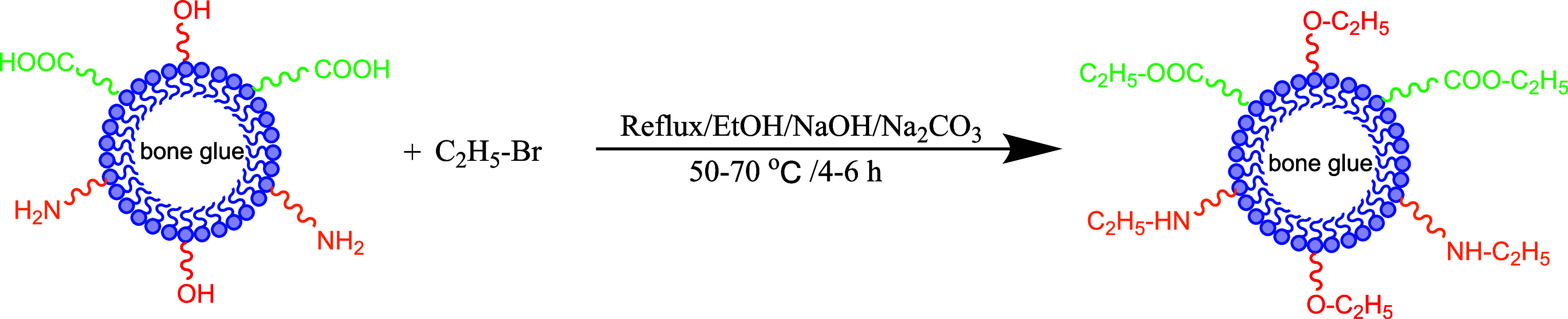
Reaction formula for BG.

**Figure 3:**
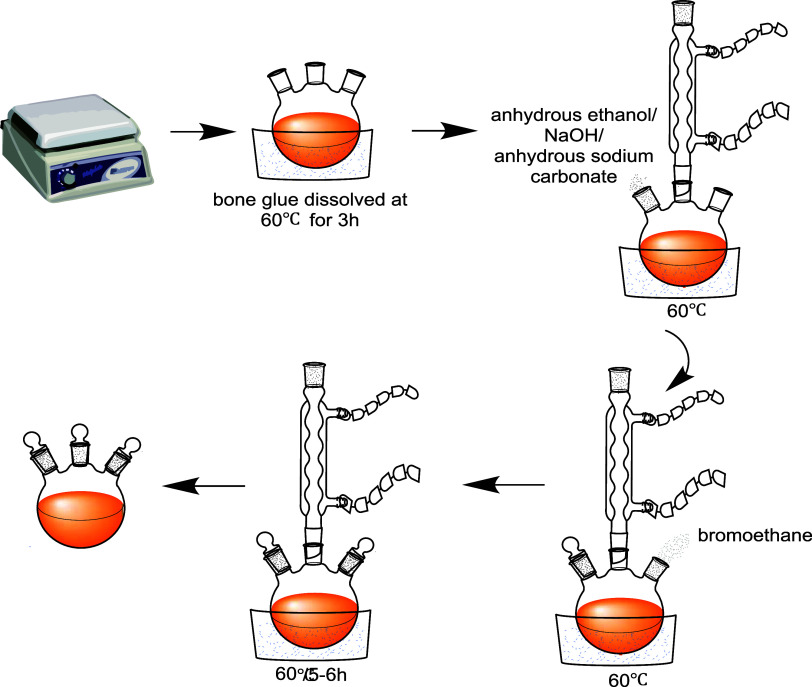
Preparation process diagram of the BG.

An appropriate amount bovine bone glue and distilled water (mass ratio of two materials was 1:1) were weighed, and then were placed in a three-necked flask, and the flask was placed in a water bath at 60 °C to make the bone glue was dissolved in distilled water for 3 h. Twenty milliliters of anhydrous ethanol, 1.0 g of sodium hydroxide, and 0.5 g of anhydrous sodium carbonate were weighed and the three drugs were added sequentially to a three-necked flask; then, the reaction was stirred at medium speed. Moreover, 4.0 g of ethyl bromide was weighed and added to the reaction during stirring; then, the reaction was refluxed for 5.5 h at 60 °C. After reaction, the substance was taken out of the flask and it was ethidium bromide-modified bone glue (BG). Figure 2 shows the reaction formula for the BG. Figure 3 displays the preparation process diagram of the BG.

### 2.3 Characterization

The sample with KBr was mixed and ground into powder for infrared spectrum (Fourier transform infrared (FT-IR)) analysis and examen by Nicolet 5700 Fourier transform infrared spectrometer (Thermoelectric Co., Ltd.) in the wavenumber range of 4000–500 cm^–1^. The scanning electron microscope (SEM) image of BG was analyzed using FEI Quanta 450 scanning electron microscope (Electron, Japan).

### 2.4 Inhibition Performance Evaluation

#### 2.4.1 Rheological Testing of Drilling Fluids

The rheological testing of drilling fluids was measured by a six-speed rotational viscometer, which can be calculated by eqs 1–3.
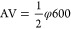
1
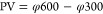
2
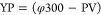
3where AV is the apparent viscosity of basic slurry (mPa·s); PV is the plastic viscosity of basic slurry (mPa·s); and YP is the yield point of basic slurry (Pa).

#### 2.4.2 Filter Loss Reduction Experiments

Six sets of 350 mL of basic slurry were prepared and 0–5% BG was added, respectively; then, the effect of BG concentration on the filtration loss of basic slurry was evaluated. Filtration loss was tested by a quadruple medium-pressure filtration loss meter and the pressure was 0.69 MPa; then, the volume of filtrate at 450 s was the filtration loss.

#### 2.4.3 Linear Swelling Experiments

1–5% BG was added to the basic slurry, respectively. 8.0 g of sodium bentonite was weighed; the core was made and their thickness was measured. The linear swelling rate was calculated by eq 4.
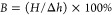
4where *B* is the linear swelling rate of basic slurry (%); *H* is the clay expansion (mm); and Δ*h* is the core thickness (mm).

### 2.5 Inhibition Mechanism Analysis

#### 2.5.1 Infrared Spectrum (FT-IR) Analysis

Two sets of 350 mL of basic slurry were prepared: one with 0% BG added and the other with 4% BG added. After the solutions were centrifuged for 15 min, then the supernatant was removed. After the remaining slurry was desiccated in an oven at 105 ± 1 °C for 24 h, the sample was prepared. The sample was mixed with KBr and ground, then placed on the Nicolet 5700 FT-IR analysis.

#### 2.5.2 Thermogravimetric Analysis (TGA)

Two sets of 350 mL basic slurry were prepared, and 0 and 4% BG were added to the two sets of basic slurry. After the solutions were centrifuged for 15 min, then the supernatant was removed. After the remaining slurry was desiccated in an oven at 105 ± 1 °C for 24 h, the sample was obtained. The thermogravimetric analysis of sample was done by using a thermogravimetric analyzer, and the parameters were set to a nitrogen flow rate of 10 mL/min and a heating rate of 20 °C/min.

#### 2.5.3 X-ray Diffraction (XRD) Analysis

Two sets of 350 mL basic slurry were prepared: one with 0% BG added and the other with 4% BG added. After the solutions were centrifuged for 15 min, the supernatant was removed. After centrifugation, the remaining slurry was dried in an oven at 105 ± 1 °C for 24 h. After drying, the sample was ground, sieved, and tested using an X-ray polycrystalline diffractometer. The experimental parameters are a scanning range of 5–80° and a scanning rate of 10°/min.

#### 2.5.4 ζ Potential Analysis

Two sets of 350 mL basic slurry were prepared: one with 0% BG added and the other with 4% BG added. Then, the two solutions were centrifuged for 15 min and the supernatant liquid was removed. Thus, the samples were prepared. The average particle size after aging the basic slurry for 16 h before and after the addition of BG was determined by the Omni multiangle particle size and a high-sensitivity ζ potential analyzer (Brookhaven), and the effect of BG on the particle size of the drilling fluids was analyzed.

## 3 Results and Discussion

### 3.1 Characterization

**Figure 4:**
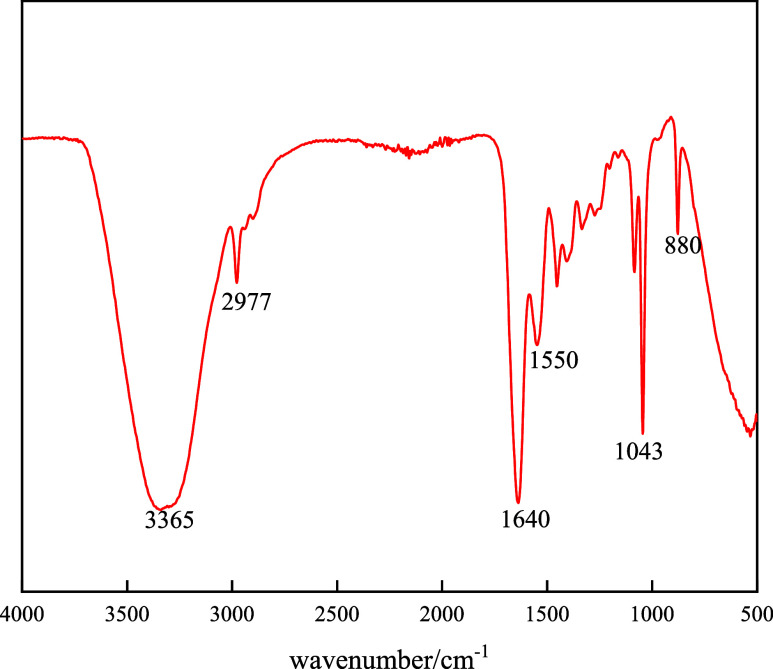
FT-IR spectra of the BG.

The structure of BG was clarified by FT-IR spectral analysis, and the results are shown in Figure 4.

As shown in Figure 4, near 3400 cm^–1^ is seen a shoulder peak and a secondary amine bending vibration appears at 1550 cm^–1^, which indicates the existence of N–H. At 2977 cm^–1^ is seen the presence of C–CH_3_. At 1640 cm^–1^ appears the C–N stretching vibrational peak. The results showed that the amino groups on animal bone glue molecules undergo a halogenation reaction with bromoethane and new products are generated. At 1043 cm^–1^ is the C–O telescopic vibrational peak. At 880 cm^–1^, the surface exhibited a (CH_2_)*_n_* plane rocking vibration. These results indicate that the animal bone glue molecules have not been broken down into small molecules during the reaction with bromoethane; thus, the goal of combining bromoethane molecules with animal bone glue molecules was achieved. The results demonstrated that the synthesized product was identical to the target molecule.

### 3.2 Inhibition Performance Evaluation

#### 3.2.1 Rheological Testing of Drilling Fluids

**Figure 5:**
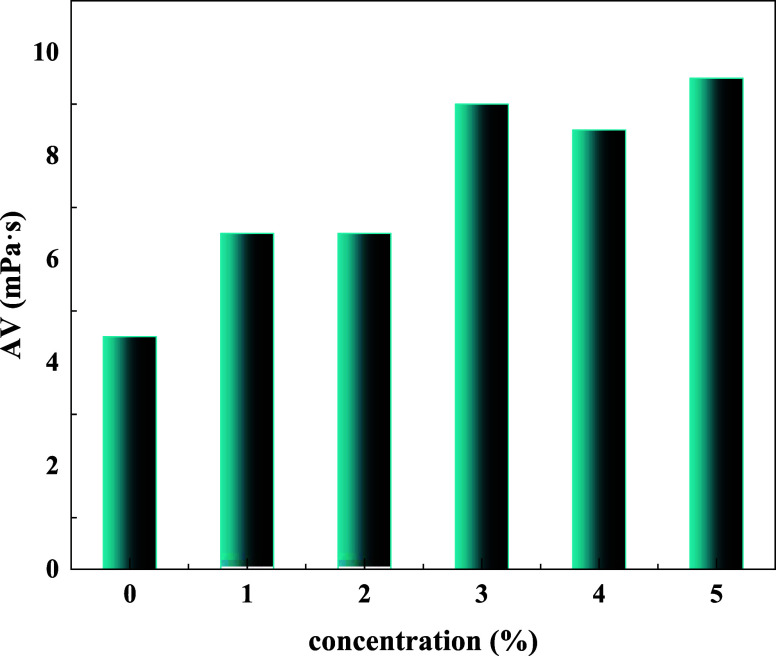
Effect of the BG concentration on apparent viscosity.

The influence of the BG concentration on the rheological properties of drilling fluids was studied after aging at 120 °C for 16 h. Results are shown in Table 1 and Figure 5.

**Table 1:** Effect of BG Concentration on the Rheological Properties of Drilling Fluids

BG concentration (%)	AV (mPa·s)	PV (mPa·s)	YP (Pa)	YP/PV (Pa/mPa·s)
0	4.5	4.0	1.0	0.25
1	6.5	6.0	1.0	0.17
2	6.5	6.0	1.0	0.17
3	9.0	8.0	1.0	0.13
4	8.5	6.0	5.0	0.83
5	9.5	8.0	3.0	0.38

As shown in Table 1, when 1–5% BG was added, the apparent viscosity of drilling fluids was increased than before. When the BG concentration was 4%, the dynamic plastic of drilling fluids ratio was higher than others, at which point the shear dilution of drilling fluids was the strongest, Compared to other concentrations, the drilling fluids with 4% BG added have the greatest ability to carry rock cuttings. The results indicated that the rheological properties of drilling fluids can be increased due to BG’s bonding abilities.

Figure 5 shows the effect of BG concentration on the apparent viscosity of basic slurry.

#### 3.2.2 Filter Loss Reduction Experiments

**Figure 6:**
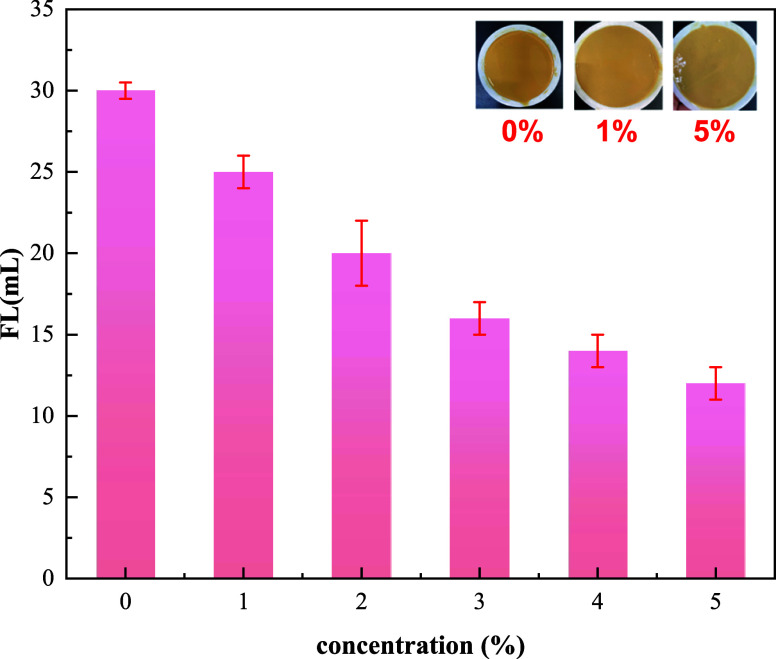
Effect of the BG concentration on filtration loss.

In this section, the effect of the BG concentration on the filtration loss reduction performance was evaluated after aging at 120 °C for 16 h. The results are displayed in Figure 6.

As shown in Figure 6, before BG was added, the base slurry’s filtration loss was 30 mL. When BG was added, the basic slurry’s filtration loss was decreased, and when 5% BG was added, its filtration loss was 12 mL, reduced by 18 mL. The results indicated that BG has a superior filtration loss reduction performance.

**Figure 7:**
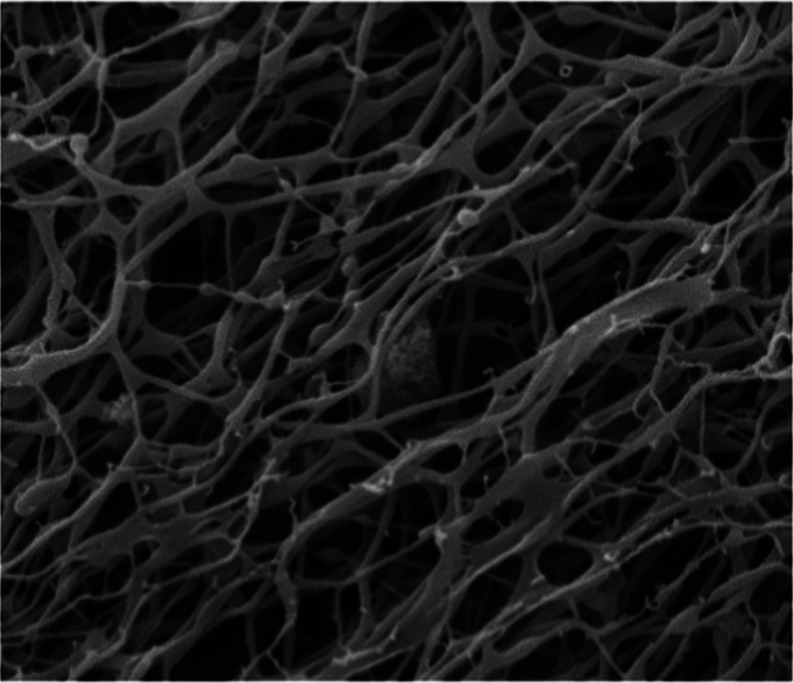
SEM image of the BG.

The BG microstructure was viewed with a scanning electron microscope, as shown in Figure 7.

In Figure 7, the BG surface shows a dense spatial network structure. It can also be seen that many branching structures were distributed on the surface of the pores between the lattices. It was concluded that the stability of BG was enhanced due to these branching structures.

#### 3.2.3 Linear Swelling Experiments

**Figure 8:**
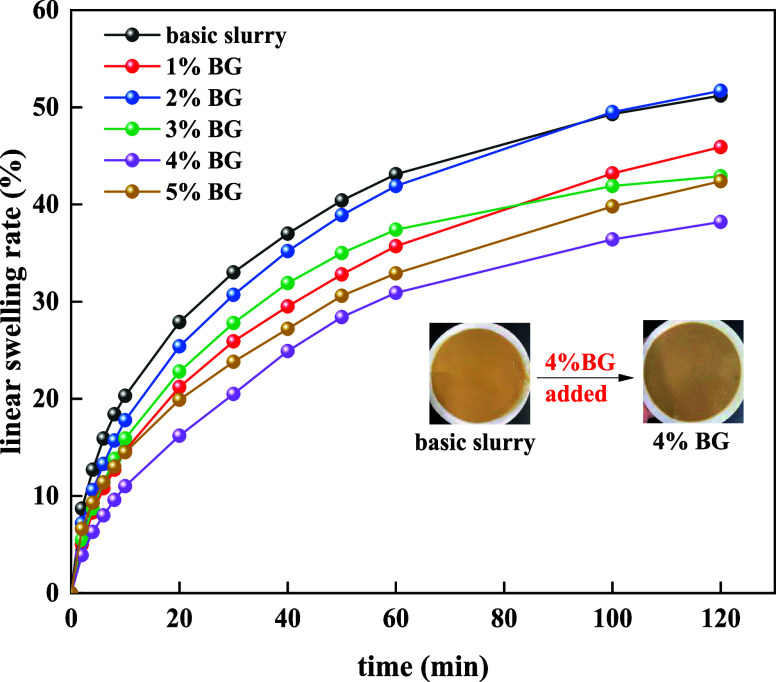
Effect of BG concentration on the linear swelling rate.

The inhibition performance of basic slurry was studied by testing the linear swelling rate at different BG concentrations. The results are shown in Figure 8.

As shown in Figure 8, at 120 °C, the linear swelling rate of basic slurry was decreased when BG was added. When 4% BG was added, the linear swelling rate of basic slurry was decreased from 50.2 to 38.2%. The results demonstrated that BG has an effective inhibition performance in basic slurry.

**Figure 9:**
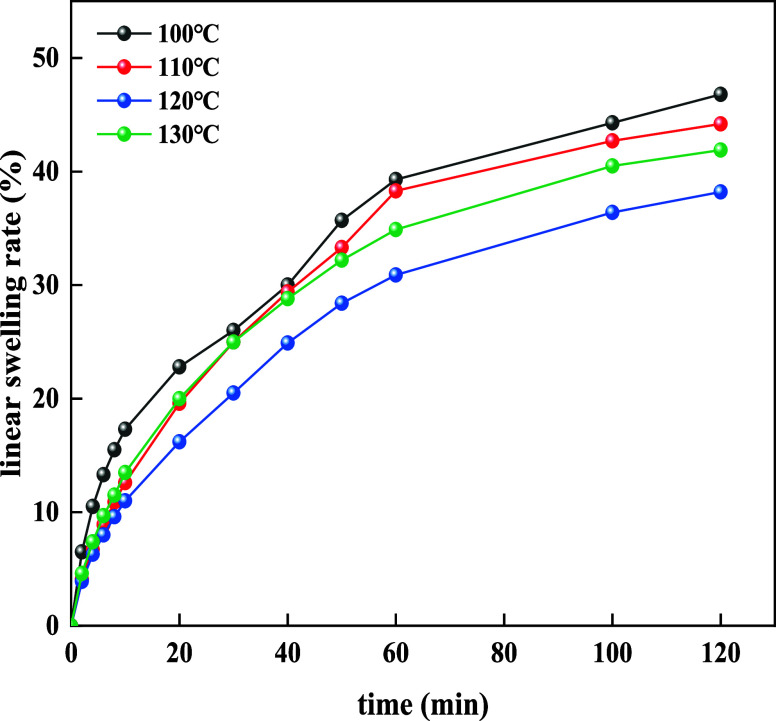
Influence of the temperature on the linear swelling rate at 4% BG.

The temperature resistance of 4% BG was evaluated, and the results are shown in Figure 9.

As shown in Figure 9, at 100 °C, the linear swelling rate of basic slurry was 25.6%. At 110 °C, the linear swelling rate of basic slurry was 30.7%. At 120 °C, the linear swelling rate of basic slurry was 29.9%. When the temperature was increased to 130 °C, the linear swelling rate of basic slurry was 29.5%. The results revealed that when 4% BG was added, its temperature resistance rose up to 130 °C.

### 3.3 Inhibition Mechanism Analysis

#### 3.3.1 Infrared Spectrum (FT-IR) Analysis

**Figure 10:**
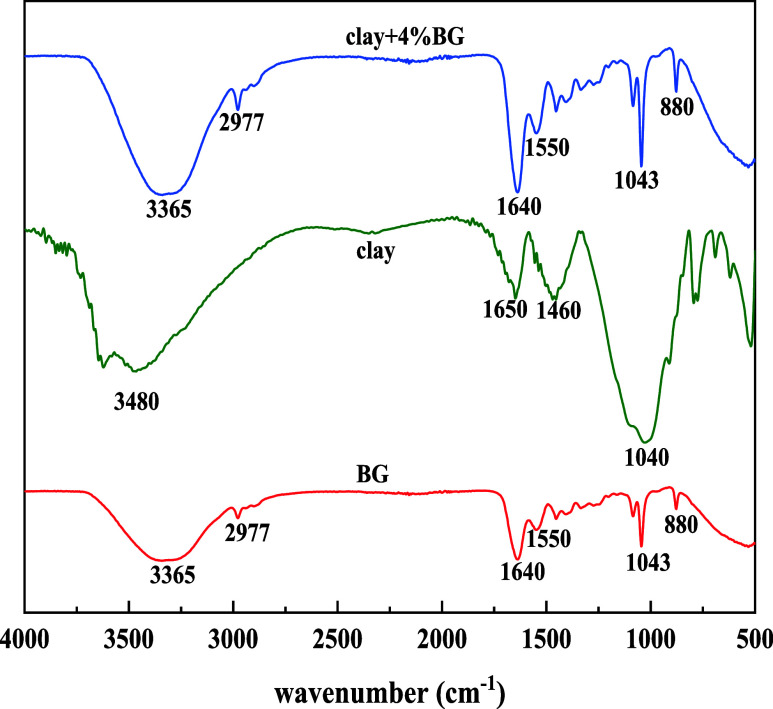
Infrared spectrum comparison.

The structure of BG, clay, and after addition of 4% BG was determined by infrared spectroscopy analysis. The results are shown in Figure 10.

As shown in Figure 10, the absorption peaks at 3365 and 1550 cm^–1^ contributed to the hydrogen bond peak of N–H, which was analyzed as the stretching vibration peak of the amino group. The absorption peaks at 2977 cm^–1^ were attributed to the C–H stretching vibration peak. The C–N stretching vibration peak appears near 1640 cm^–1^. At 1043 cm^–1^ appeared the bending vibration peaks, attributed to the C–O. At 880 cm^–1^, a weak absorption band appeared, indicating the occurrence of (CH_2_)*_n_* plane rocking vibration and showing that animal bone glue molecules did not decompose into small molecules during the reaction with bromoethane, achieving the goal of combining bromoethane molecules with animal bone glue molecules. The results signified that after BG was added, it was absorbed in the clay and the structure stability of the clay was enhanced.

#### 3.3.2 Thermogravimetric Analysis (TGA)

**Figure 11:**
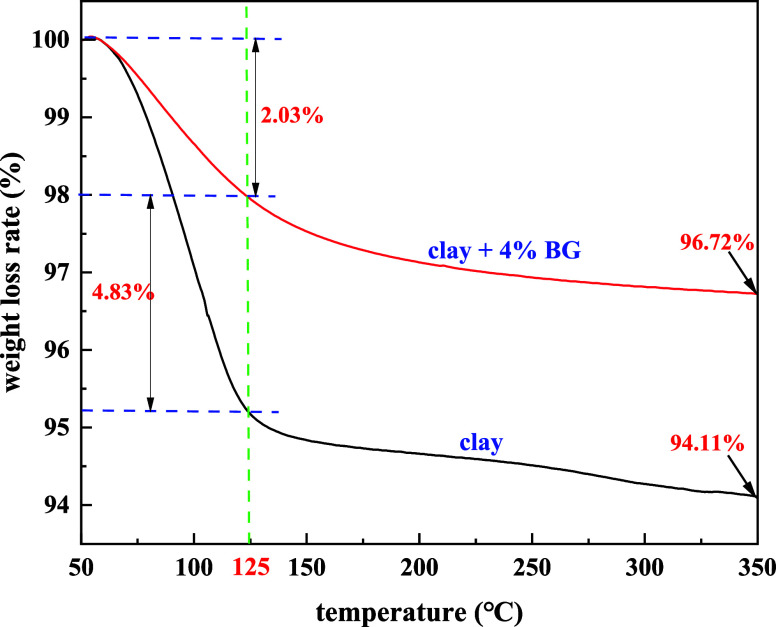
Thermogravimetric analysis chart.

The temperature resistance of BG was studied by thermogravimetric analysis. Figure 11 shows the thermogravimetric analysis comparison chart.

As shown in Figure 11, the clay’s quality was lost between 50 and 125 °C, with a mass loss of 4.83%. When 4% BG was added, the mass loss was 4.23%, and it was analyzed that this is a process of water evaporation. At 125–350 °C, the sample’s quality continued to decrease and it was inferred that its structure had changed.

#### 3.3.3 X-ray Diffraction (XRD) Analysis

**Figure 12:**
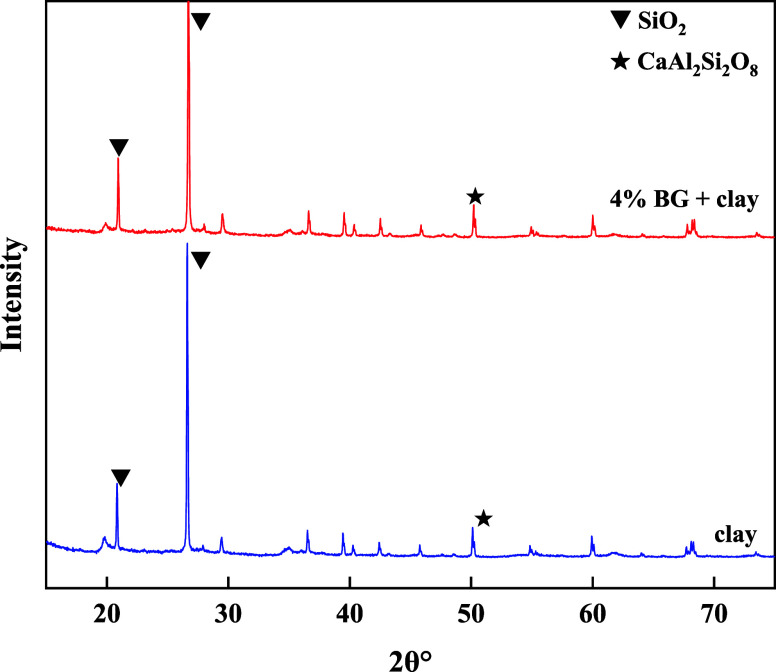
XRD analysis comparison chart.

The component of clay before and after 4% BG was identified by X-ray diffraction (XRD) analysis. The results are illustrated in Figure 12.

As can be seen from Figure 12, SiO_2_ characteristic peaks appeared at 21 and 27°, and at 50° the CaAl_2_Si_2_O_8_ characteristic peak. Compared with the basic slurry, the characteristic peaks appearing after the addition of 4% BG were basically the same in both cases. Compared with the XRD spectra of basic slurry before and after the addition of 4% BG, it was found that the corresponding peaks were observed and became smaller, which suggested indirectly that the main functional groups of BG were adsorbed in the clay and the adsorption effect was significant. The results suggested that the synthesis of BG was successful.

### 3.4 ζ Potential Analysis

**Figure 13:**
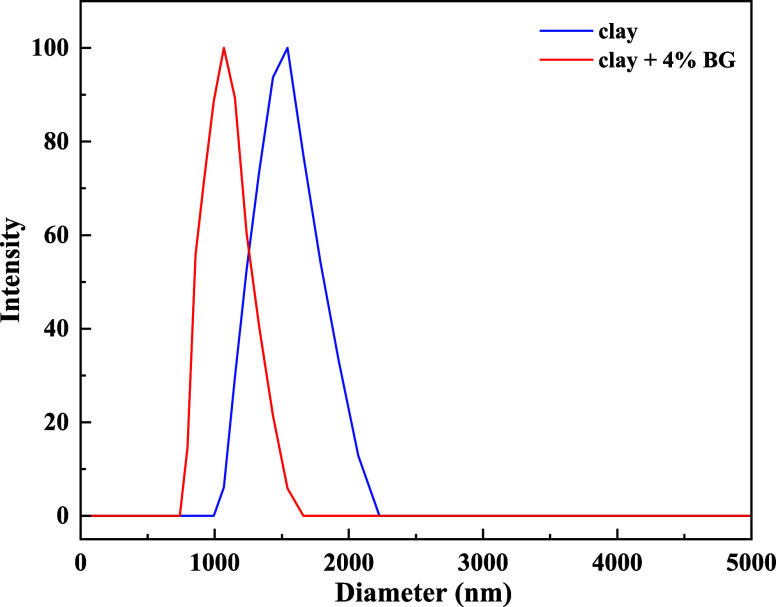
Particle size analysis comparison chart.

The average particle size of the clay before and after addition of 4% BG was studied by ζ potential analysis. Figure 13 shows the clay’s particle size analysis before and after the addition of 4%BG.

As shown in Figure 13, the average particle size of clay was 1.251 μm; when 4% BG was added, the average particle size of clay declined to 0.749 μm. The results suggested that the average particle size of clay was decreased with addition of BG, revealing that BG provided better dispersibility and could avoid agglomeration and precipitation.

**Figure 14:**
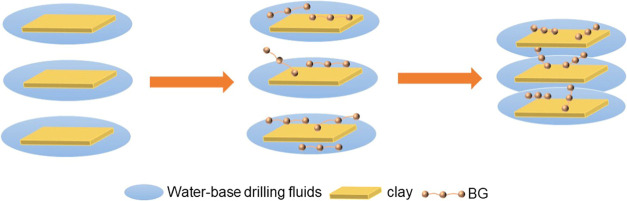
Inhibition mechanism of BG.

As shown in Figure 14, the inhibition mechanism of BG was that many reactive functional groups exist in BG’s molecular structure, which undergo chemical cross-linking by functional groups such as hydroxyl and carboxyl groups. Based on this, some new functional bonds were generated within its molecular structure and adsorbed on the surface of clay, thereby inhibiting clay swelling.

## 4 Conclusions

An environmentally friendly water-based drilling fluid additive BG was developed using animal bone glue and bromoethane as raw materials, anhydrous ethanol as the solvent, and sodium hydroxide as the alkaline hydrolysis agent; anhydrous sodium carbonate regulated the pH of the system solution.

(1) Different concentrations of BG were added into the basic slurry, and its performance in drilling fluids was investigated by linear swelling experiments and drilling fluids performance evaluation experiments. When 4% BG was added at 120 °C, the linear swelling rate decreased from 50.2 to 38.2%. When 5% BG was added, the filtration loss decreased from 30 to 12 mL during the drilling fluids performance evaluation and showed an excellent filtration loss reduction impact. The temperature resistance of BG was investigated; the results showed the temperature resistance of BG of up to 130 °C. Based on this, the results indicated that BG has better temperature resistance and inhibition. The microstructure of BG was observed by scanning electron microscopy (SEM); BG’s structure was stable.
(2) The inhibition mechanism of BG in the drilling fluids was studied by infrared spectrum analysis, thermogravimetric analysis, X-ray diffraction analysis, and ζ potential analysis. The effective synthesis of BG and the adsorption of its main functional groups in the drilling fluids were seen by the infrared spectrum and X-ray diffraction patterns. When 4% BG was added in basic slurry, its thermal stability was better than that of basic slurry as observed by thermogravimetric analysis. Through the ζ potential analysis, we observed that the particle size of clay was decreased by 0.502 μm, from 1.251 to 0.749 μm. Based on these results, we concluded that BG has good filtration and inhibition properties.

## Data Availability

All data generated or analyzed during this study are included in this published article.
